# Diphtheria

**Published:** 1881-01

**Authors:** Ad. Lippe


### REVIEWS.

"Diphtheria," by Rollin R. Gregg, M.D., Buffalo, 1880.—Here is presented to the profession a truly Homœopathic book. It deserves to be read and used by every Homœopathic healer; it shows that its author has followed Hahnemann's teachings carefully and he confirms the wise admonitions of that close observer. Hahnemann advised the healer as to what was to be cured, how the remedy should be selected and how it should be administered. Dr. Gregg followed this advice and found that Hahnemann was right when he said in the 154th paragraph of the "Organon," "a disease which is of no very long standing ordinarily yields, without any degree of suffering, to a first dose of this medicine." The indications for the various medicines in Diphtheria are clearly given, they have been so often confirmed that they leave no possible doubt as to their reliability. The only addition we may ask to be made is that the characteristic symptom of *Lachesis* "worse after sleep" may also be added to *kali bich.*; though found from clinical observations, it nevertheless is a reliable symptom. Dr. Gregg shows clearly that there can never be found a specific remedy for a specific disease; but that we can and must find for each and every individual case the similar remedy and when found administer it in the single dose.—Ad. Lippe.

